# Integrative Molecular, Cytological, and Anatomical Analyses Reveal Two Distinct Clusters in *Fittonia* Cultivars

**DOI:** 10.3390/plants15091391

**Published:** 2026-05-01

**Authors:** Min Deng, Qiansheng Li, Jianjun Chen

**Affiliations:** 1 Mid-Florida Education and Research Centre, Horticultural Sciences Department, Institute of Food and Agricultural Sciences, University of Florida, 2725 Binion Road, Apopka, FL 32703, USA; dengmin@ynu.edu.cn (M.D.); qli@atlantabg.org (Q.L.); 2 State Key Laboratory of Vegetation Structure, Function and Construction (VegLab); Ministry of Education Key Laboratory for Transboundary Ecosecurity of Southwest China; Yunnan Key Laboratory of Biological Adaptation, Conservation and Utilization; School of Ecology and Environmental Science, Yunnan University, Kunming 650500, China; 3 Southeastern Center for Conservation, Atlanta Botanical Garden, 1345 Piedmont Avenue NE, Atlanta, GA 30309, USA

**Keywords:** AFLP, *Fittonia*, flow cytometry, MSAP, ornamental foliage plants

## Abstract

*Fittonia* Coem. is a widely cultivated ornamental foliage genus, but the taxonomy and relationships of cultivated forms remain poorly resolved because traditional classifications rely heavily on variable foliar traits. In this study, we investigated the genetic relationships and phenotypic variation among 14 commercially available *Fittonia* cultivars using amplified fragment length polymorphism (AFLP), methylation-sensitive amplified fragment length polymorphism (MSAP), DNA flow cytometry, chromosome counting, and comparative morphological and anatomical analyses. Morphological character states were further mapped onto AFLP- and MSAP-derived phylograms to assess their evolutionary patterns and taxonomic relevance. AFLP and MSAP analyses consistently recovered two well-supported clusters within the cultivated material examined: Clade I, comprising ‘Titanic’ and ‘Angel Snow’, and Clade II, comprising the remaining 12 cultivars. These clades were independently supported by stable differences in trichome morphology, cystolith distribution, epidermal cell form, leaf venation architecture, and stem anatomy. In contrast, several traditionally used diagnostic characters, including leaf vein color and petiole length, were found to be homoplastic or continuously variable. Chromosome counts and flow cytometry indicated that all cultivars were diploid (2*n* = 36) and exhibited limited variation in genome size. Together, these results provide integrative evidence for two distinct clusters within cultivated *Fittonia*. However, because sampling was limited to commercially available cultivars, the relationship of these clusters to currently recognized species remains unresolved. Our findings highlight the limitations of traditional trait-based classifications and underscore the need for broader sampling, including wild populations, to reassess species boundaries in the genus.

## 1. Introduction

*Fittonia* Coem. is a small genus of tropical perennial herbs native to Colombia, Ecuador, Peru, and northern Brazil [1,2]. For their striking foliage, marked by networks of white, pink, or red veins, *Fittonia* species are widely cultivated as ornamental foliage plants. They are commonly known as nerve plants or silver-nerve plants. The horticultural history of the genus dates to the 1860s, when *Fittonia albivenis* (Veitch) Brummitt and *F. gigantea* Linden were introduced into England and Belgium and subsequently distributed throughout Europe [2,3]. Over more than 150 years of introduction, vegetative propagation, and horticultural selection, numerous cultivars differing in leaf size, shape, color, and variegation patterns have been developed. Commercially important cultivars include Angel Snow, Frankie, Fortissima, Black Star, Jacmita, Leather Leaf, Mini-Josan, Red Angel, Red Anne, Red Star, Red Vein, Superba, Titanic, and White Anne, all of which are now widely produced as indoor foliage plants worldwide [4]. In addition to their ornamental value, recent studies have shown that *Fittonia* can respond efficiently to far-red light and that genomic resources are emerging, including a near-complete haplotype-resolved genome and regulatory analyses of chlorophyll and anthocyanin biosynthesis [5,6,7].

Despite its horticultural importance and increasing research attention, the taxonomy of *Fittonia* remains unresolved. Early classifications recognized three species: *F. argyroneura* Coemans, *F. gigantea* Linden, and *F. albivenis* (Lem.) Coemans, and additional infraspecific distinctions were proposed based largely on leaf venation color, plant stature, and petiole length. For example, within *F. argyroneura*, the white-veined cultivar Argyroneura and the more robust Pearcei were distinguished by DeWolf [1] and in Hortus Third [8]. Later treatments reduced the genus to two species, *F. gigantea* and *F. albivenis*, with *F. verschaffeltii* treated as a synonym [1,2,3]. In that framework, *F. gigantea* was defined primarily by its larger habit, longer petioles, and white-veined leaves, whereas *F. albivenis* included smaller, creeping plants with shorter petioles. Brummitt [3] further subdivided *F. albivenis* into two informal groups, the “Argyroneura group” and the “Verschaffeltii group,” largely on the basis of venation color. However, these classifications are largely based on cultivated material and a limited set of morphological traits.

The difficulty with these classifications is that the characters on which they rely are highly plastic, continuous, or horticulturally selected. Leaf venation color, degree of variegation, blade size, and growth habit are all traits that can be strongly modified by selection and may not faithfully track underlying evolutionary differentiation. As a result, morphological resemblance among cultivars may reflect convergence or parallel selection for ornamental traits rather than shared ancestry. Conversely, less conspicuous structural traits, such as leaf architecture, epidermal micromorphology, trichome type, and cystolith distribution, may be more conserved and therefore more informative for taxonomic purposes. This raises a central systematic question: do currently cultivated *Fittonia* represent variation within a single highly polymorphic taxon, or do they reflect multiple distinct evolutionary groups or lineages obscured by overlapping horticultural phenotypes?

Previous anatomical and cytological observations suggest that the latter possibility deserves serious consideration. Ahmad [9] reported that the dense ocelli in ‘Pearcei’ differed from those of other taxa and proposed that it might represent a distinct species. Cytological studies also suggested ploidy variation, reporting triploidy in ‘Pearcei’ (2*n* = 54) and diploidy in var. verschaffeltii, var. argyroneura, and *F. gigantea* (2*n* = 36) [10,11]. At the same time, the overlap and inconsistency of diagnostic characters led Brummitt [3] to suggest that perhaps only a single species could be recognized in the genus. Thus, both lumping and splitting interpretations have persisted, and relationships among cultivated forms remain uncertain. Importantly, most of these interpretations are based on limited samples of cultivated materials rather than on comprehensive surveys of natural populations.

Resolving this uncertainty requires an integrative approach. Marker systems such as amplified fragment length polymorphism (AFLP) can detect broad genomic differentiation among closely related taxa [12], whereas methylation-sensitive amplified fragment length polymorphism (MSAP) can reveal epigenetic divergence associated with cytosine methylation patterns. When combined with flow cytometry, chromosome counts, and comparative anatomy, these methods can help distinguish patterns of differentiation associated with major genomic or cytological differences from those arising primarily through structural or regulatory variation. Such integration is particularly important in *Fittonia*, where striking foliar diversity may reflect relatively limited genomic divergence but substantial developmental and epigenetic differentiation.

The objectives of this study were therefore to clarify the relationships among currently cultivated *Fittonia* using AFLP, MSAP, DNA flow cytometry, chromosome counting, and traditional morphological and anatomical analyses. In addition, morphological character states were mapped onto AFLP- and MSAP-derived phylogenetic trees to assess whether historically important taxonomic characters reflect shared ancestry or repeated, independent evolution. By combining genomic, epigenetic, cytological, and anatomical evidence, we sought to determine whether cultivated *Fittonia* represents a single variable cluster or multiple distinct lineages and to identify characters that may provide a more reliable basis for future systematic assessment. This study provides an important framework for interpreting phenotypic variation and offers useful insights into the classification, trait-based selection, and breeding of ornamental *Fittonia* cultivars.

## 2. Materials and Methods

### 2.1. Plant Materials

A total of 14 *Fittonia* cultivars and two outgroup taxa, *Aphelandra squarrosa* Nees ‘Dania’ and *Hypoestes* ‘Pink Splash’ (Acanthaceae), were collected from research greenhouses at the University of Florida Mid-Florida Research and Education Center (MREC) and from commercial ornamental foliage plant nurseries in central Florida (Table S1).

Each cultivar was represented by a single horticultural accession maintained under greenhouse conditions, reflecting typical clonal propagation practices in cultivated *Fittonia*. Because cultivars are vegetatively propagated, within-cultivar genetic variation is expected to be minimal; the sampling design therefore focuses on comparisons among cultivars rather than on population-level variation. Sampling was restricted to commercially available cultivars from a limited geographic source; thus, the results are intended to reflect patterns within cultivated material rather than the full diversity of the genus. Voucher specimens were deposited in the Florida Museum of Natural History Herbarium (FLAS).

### 2.2. Molecular Analysis

#### 2.2.1. DNA Extraction

Fresh young leaves were collected and immediately frozen in liquid nitrogen. Total genomic DNA was extracted using the cetyltrimethylammonium bromide (CTAB) method as described by Bousquet et al. [13]. DNA quality was assessed by electrophoresis on 0.65% agarose gels. DNA concentration was measured using the Turner Biosystems TB5-380 fluorometer (Turner BioSystems Inc., Sunnyvale, CA, USA).

#### 2.2.2. Amplified Fragment Length Polymorphism (AFLP) Analysis

AFLP analysis was performed following the protocol of Vos et al. [14] with minor modifications. Restriction and ligation reactions were conducted in separate steps. For restriction digestion, 200 ng of genomic DNA from each sample was digested with 2 U of *Eco*RI and 2 U of *Mse*I (New England Biolabs, Ipswich, MA, USA) at 37 °C overnight in 20 µL reaction volumes containing sterile water, 0.1% bovine serum albumin (BSA), and 10× NEBuffer 2.

Ligation reactions were performed in 20 µL volumes containing 1 U of T4 DNA ligase (Promega, Madison, WI, USA), 2 µL of 10× ligase buffer, 0.5 µM *Eco*RI adapter, 5 µM *Mse*I adapter, 5 µL of digested DNA, and sterile water. Reactions were incubated at 20 °C for 12 h.

Pre-amplification reactions were carried out in 20 µL volumes containing 2 µL of restriction–ligation product, 1 U Taq DNA polymerase (Promega), 5× PCR buffer, 3.5 mM MgCl_2_, 0.5 mM dNTPs, and 1.25 ng µL^−1^ each of *Eco*RI + A and *Mse*I + C primers. Amplification was performed using a Bio-Rad DNAEngine^®^ PCR machine (Bio-Rad Laboratories, Hercules, CA, USA) under the following conditions: 1 cycle at 94 °C for 1 min; 20 cycles of 94 °C for 30 s, 56 °C for 1 min, and 72 °C for 1 min; followed by a final extension at 72 °C for 10 min. Pre-amplified products were diluted 1:20 with double-distilled water and used as templates for selective amplification.

Selective amplification was conducted in 12 µL reaction volumes containing 3 µL diluted pre-amplification product, 0.5 U Taq DNA polymerase (Promega), 5× PCR buffer, 3 mM MgCl_2_, 0.25 mM dNTPs, 0.05 µM IRDye700- or IRDye800-labeled *Eco*RI + 3 primers, and 0.125 µM *Mse*I + 3 primers. The cycling profile consisted of 94 °C for 2 min; 10 cycles of 94 °C for 30 s, 65 °C for 30 s (decreasing by 0.7 °C per cycle), and 72 °C for 60 s; followed by 23 cycles of 94 °C for 30 s, 56 °C for 30 s, and 72 °C for 60 s; with a final extension at 72 °C for 5 min.

Five mL of Blue Stop loading buffer (LI-COR, Lincoln, NE, USA) was added to each reaction. Samples were denatured at 94 °C for 3 min, chilled on ice, and immediately loaded onto a 25 cm × 0.25 mm 6.5% denaturing polyacrylamide gel. Electrophoresis was performed in 1× TBE buffer using a LI-COR 4300 DNA Analyzer (LI-COR, Inc.). Twelve *Eco*RI + 3 and *Mse*I + 3 primer combinations were analyzed (Table S2).

#### 2.2.3. Methylation-Sensitive Amplified Fragment Length Polymorphism (MSAP) Analysis

MSAP analysis followed the AFLP protocol, with modifications to the restriction enzymes. *Mse*I was replaced by the methylation-sensitive isoschizomers *Hpa*II and *Msp*I. The *Eco*RI adapters and primers remained unchanged, whereas adapters and primers for *Hpa*II and *Msp*I were designed according to Salmon et al. [15,16] (Table S2). Restriction–ligation reactions and PCR amplification conditions were identical to those used for AFLP.

### 2.3. Data Analysis

*Hpa*II and *Msp*I recognize the same restriction site (5′-CCGG) but differ in sensitivity to cytosine methylation. *Hpa*II is sensitive to methylation of the internal cytosine on both strands, whereas *Msp*I is sensitive to methylation of the external cytosine. Fragments present in both *Eco*RI/*Hpa*II and *Eco*RI/*Msp*I digests were classified as type I (non-methylated). Fragments presented only in *Eco*RI/*Msp*I digests were classified as type II (fully methylated internal cytosines). Fragments present only in *Eco*RI/*Hpa*II digests were classified as type III (hemimethylated). Fragments absent in both digests were classified as type IV (fully methylated) [17].

AFLP and MSAP fragments were scored using Saga™ Automated AFLP Analysis Software version 3.3 (LI-COR, Inc.) and verified by visual inspection. Bands were scored as binary characters: present (1) or absent (0). Fragments showing differences in intensity or presence/absence between *Eco*RI/*Hpa*II and *Eco*RI/*Msp*I digests were treated as methylation-sensitive polymorphisms following Cervera et al. [18]. Fragment types (I–IV) were annotated for each genotype.

AFLP and MSAP binary matrices were analyzed separately and in combination to assess clustering stability and consistency. Because many AFLP markers were autapomorphic and could produce long-branch artifacts, distance-based Neighbor-Joining analyses using Nei–Li distances were performed in PAUP* 4.0b10 [19]. Monomorphic bands were excluded. *Aphelandra squarrosa* was used as the outgroup to root the trees. Bootstrap support was evaluated using 50,000 replicates.

Bayesian inference was performed using MrBaryes 3 [20,21] to estimate support for clustering patterns. The restriction-site (binary) model was used with the default Markov chain Monte Carlo (MCMC) settings. Analyses were run for 1,000,000 generations, with trees sampled every 1000 generations. Convergence was evaluated using Tracer v1.6 [22], and the first 15% of sampled trees were discarded as burn-in after confirming stabilization of log-likelihood values.

### 2.4. Cytological Analysis

#### 2.4.1. Chromosome Analysis

Root tips were collected from plants in the morning (08:30–09:00 h) and pre-treated with 2.0 mM 8-hydroxyquinoline for 3 h at room temperature. Samples were fixed in a 1:3 (*v*/*v*) mixture of glacial acetic acid and absolute ethanol for 2 h at 4 °C and then stored in 70% ethanol at −20 °C. Root tips were softened by hydrolysis in 1 N HCl at 60 °C for 5 min, briefly rinsed in distilled water, and stained with 2% aceto-orcein (Carolina Biological Supply Co. Burlington, NC, USA) for approximately 15 min.

Stained root tips were mounted on glass slides and gently macerated into small fragments using fine needles with a fresh drop of stain. Coverslips were applied and briefly warmed over an alcohol lamp. Preparations were gently tapped and squashed. Chromosome numbers were determined from 5 to 10 well-spread mitotic metaphase cells using a Nikon Optiphot Pol microscope (Nikon Instruments Inc., Melville, NY, USA). Images were captured with a Canon digital camera (Canon Inc., Tokyo, Japan).

#### 2.4.2. Flow Cytometry Analysis

The nuclear DNA content of *Fittonia* cultivars was determined using a Partec PAS flow cytometer (Partec, Münster, Germany) according to the manufacturer’s protocol with minor modifications. Young leaf tissue (0.2–0.5 cm^2^) of pea (*Pisum sativum* ‘Ctirad’) was used as an internal reference standard. Leaf tissues of the standard and each *Fittonia* sample were chopped together in individual plastic Petri dishes containing 0.5 mL extraction buffer (CyStain^®^ PI Absolute P kit, Partec, Munster, Germany) using a razor blade.

The homogenate was incubated for 0.5–1 min at room temperature and filtered through a 50 μm nylon mesh to remove debris. Two mL of staining buffer containing RNase and propidium iodide (PI) were added to the nuclei suspension, which was incubated for at least 30 min at room temperature to allow complete staining. Fluorescence intensities were measured using a flow cytometer equipped with a 488 nm argon laser and long-pass filter (Partec). Ploidy level and nuclear DNA content were estimated following Cui et al. [23]. Each sample was analyzed in three independent replicates.

### 2.5. Anatomical Analysis

The fourth fully expanded leaf from the shoot apex of each cultivar was sampled. Two 2 × 2 cm segments were excised from each leaf, one from the central region adjacent to the primary vein and the other from the marginal region. The segments were fixed in FAA (formalin: glacial acetic acid: 70% ethanol, 5:5:90, *v*/*v*). Samples were dehydrated through an ethanol–xylol series, processed using standard paraffin-embedding procedures, and embedded in Paraplast tissue embedding medium (Leica Biosystems Richmond, Inc., Richmond, IL, USA), which has a melting point of 56–58 °C.

Transverse leaf sections (7–8 µm thick) were stained with safranin–fast green. Petiole samples (0.5 cm in length) were collected from the midpoint of the petiole, and stem samples were taken from the internode between the third and fourth leaves. Petiole and stem sections were prepared following the same procedures used for leaf sections.

Cuticle, stomatal, and venation preparations followed the protocols of Stace [24,25], and leaf architecture samples were prepared following Luo and Zhou [26]. Stomatal density was determined as the mean number of stomata per unit area from five randomly selected fields under a 10× ocular lens and 10× objective lens. All transverse sections, epidermal preparations, and leaf architecture samples were examined using a Nikon OPTIPHOT microscope (Nikon Instruments Inc.) and photographed with a Canon S3 IS digital camera (Canon Inc.). All permanent slides were deposited at MREC. Anatomical terminology followed Hickey [27], Ahmad [9], Stace [28], and the Leaf Architecture Working Group [29].

### 2.6. Morphological Analysis

Five commonly used morphological characters in traditional taxonomy: (1) plant habit, (2) size of inflorescence bracteoles, (3) petiole length, (4) leaf venation color, and (5) bracteole shape, were measured from five pots of each *Fittonia* cultivar. Because bracteole size and petiole length decrease gradually from the apex to the base of the plant, only basal bracteoles and petioles from basal leaves were measured.

Character state delimitation and comparisons were based on current observations and previous studies (Table S3). Morphological character states were coded as absent (0) or present (1). Character state transitions were reconstructed on the AFLP/MSAP phylogram using MESQUITE version 2.75 [30]. These characters were used for comparative purposes to evaluate their consistency with molecular clustering patterns, rather than for formal taxonomic delimitation.

## 3. Results

### 3.1. Genetic Relationships Among Fittonia Cultivars

The 12 AFLP primer-pair combinations generated 549 clear fragments, of which 292 (53.1%) were polymorphic. In the MSAP analysis, 667 fragments were obtained from 12 primer-pair combinations, including 212 polymorphic fragments (31.8%) from *Eco*RI/*Msp*I and 265 polymorphic fragments (38.4%) from *Eco*RI/*Hpa*II. The total number of fragments and polymorphic loci detected by AFLP and MSAP is summarized in Table 1.

**Figure 1:**
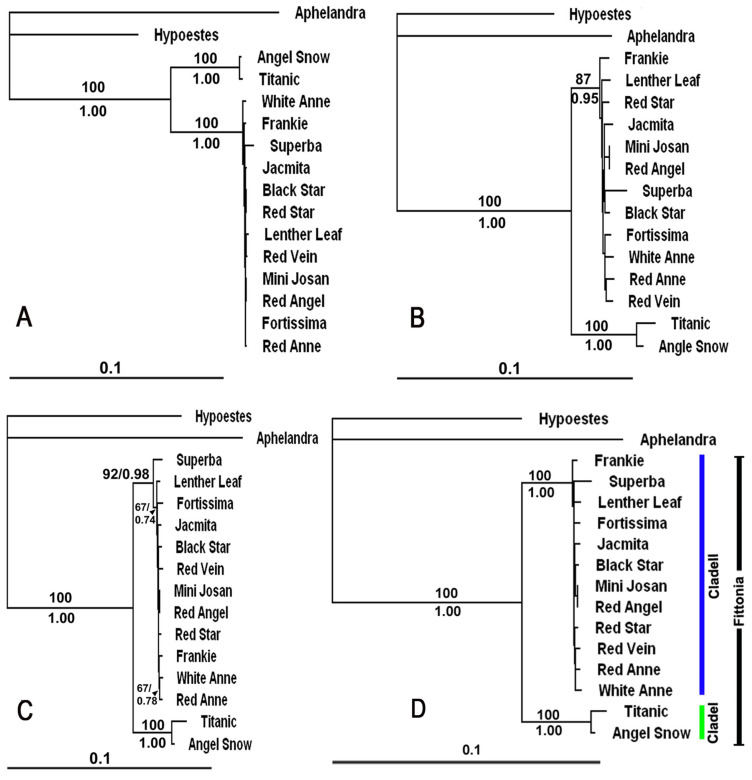
**Phylogram of 14 *Fittonia* cultivars constructed according to AFLP, MSAP, and combined datasets**. The constructions used the Neighbor-Joining Method with Nei-Li distances. *Aphelandra squarrosa* was used as the outgroup to root the tree. Values above the branch (or before the slash) indicate bootstrap support based on 10,000 nonparametric replicates calculated by PAUP 4.0b [19]. Values below branches (or after the slash) represent posterior probabilities inferred using MrBaryes 3 [21] from 1,000,000 generations. (**A**). AFLP phylogram (total branch length = 0.50760). (**B**). *Msp*I-based MSAP phylogram (total branch length = 0.32075). (**C**). *Hpa*II-based MSAP phylogram (total branch length = 0.32282). (**D**). Combined AFLP and MSAP phylogram (total branch length = 0.32616).

Neighbor-joining analysis based on Nei–Li genetic distances produced an AFLP tree with a total branch length of 0.50760 (Figure 1A), an *Msp*I-MSAP tree with a total branch length of 0.32075 (Figure 1B), and an *Hpa*II-MSAP tree with a total branch length of 0.32282 (Figure 1C). The topologies of these three trees were highly congruent. The monophyly of the sampled *Fittonia* cultivars was strongly supported (100% bootstrap). Two major clusters were consistently recovered: Clade I, comprising ‘Titanic’ and ‘Angel Snow’, supported by 100% bootstrap, and Clade II, including the remaining 12 cultivars, supported by 87–100% bootstrap values. Within Clade II, no internal nodes were supported by bootstrap values > 80% in either the AFLP or *Hpa*II-MSAP analyses, and only two weakly supported nodes (< 80%) were observed in the *Msp*I-MSAP phylogram.

Combined analysis of the three datasets produced a tree topology (Figure 1D) similar to those derived from AFLP and MSAP alone, with strong support (100% bootstrap) for the monophyly of *Fittonia* and for the separation into two major clusters within the cultivated material examined. However, no stable substructure within Clade II was supported (Figure 1C).

#### 3.1.1. MSAP Analysis

A total of 667 MSAP fragments were detected and classified into four fragment types (Figure S1). Type I fragments ranged from 200 to 221 across cultivars, of which approximately 30–47% were methylation-sensitive. Type II fragments (internal cytosine methylation) ranged from 181 to 216, Type III fragments (hemi-methylated cytosine) from 144 to 164, and Type IV fragments (fully methylated cytosines on both strands) from 84 to 134. The relative proportions of the four fragment types differed among cultivars (Table 2). ‘Superba’ exhibited the highest overall methylation level (82.78%), whereas ‘Angel Snow’ had the lowest (76.76%). ‘Titanic’ showed the highest proportion of fully methylated fragments (47.31%), while the lowest proportion of full methylation was observed in ‘Fortissima’ (44.46%).

Many methylated and non-methylated fragments were shared across cultivars, indicating similar patterns (Table 2). A total of 116 Type I, 84 Type II, and 77 Type III fragments were shared by all cultivars. Among polymorphic fragments, 23% of the variation was associated with the differentiation of ‘Titanic’ and ‘Angel Snow’ from the remaining cultivars. Thirty-nine Type I fragments (21.6% of all Type I fragments) were unique to ‘Titanic’ and ‘Angel Snow’, whereas 51 Type I fragments (27.2%) were present only in the remaining 11 cultivars. Similarly, 39 and 51 Type I fragments, 20 and 22 Type II fragments, and 21 and 25 Type III fragments were shared by Clade I and Clade II, respectively.

#### 3.1.2. Genome Size and Cytology of *Fittonia* Cultivars

**Figure 2:**
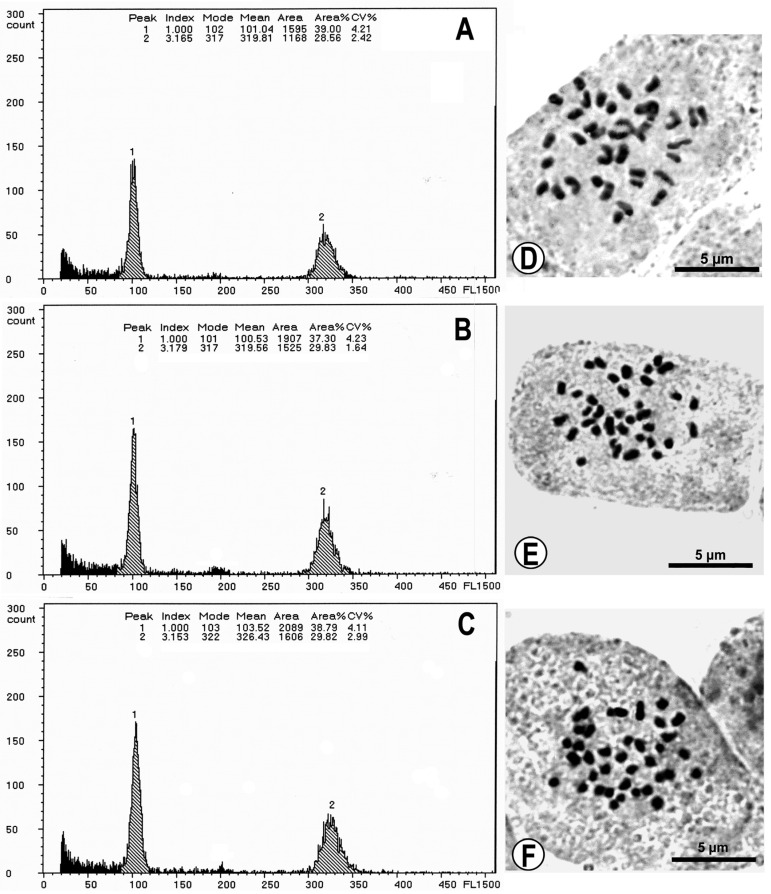
Fluorescent intensities by flow cytometry and chromosome spreads of three *Fittonia* cultivars: (**A**,**D**). Cultivar Red Anne; (**B**,**E**). Cultivar Leather Leaf; (**C**,**F**). Cultivar Titanic. (**A**–**C**). Peak 1 indicates *Fittonia* cultivars, peak 2 indicates *Pisum sativum* ‘Ctirad’. All the cultivars were diploid, with a chromosome number of 2*n* = 36.

Flow cytometric profiles of all *Fittonia* cultivars exhibited distinct and consistent fluorescence peaks (Figure 2A–C). Genome size estimates, calculated relative to the internal standard (*Pisum sativum* ‘Ctirad’), are presented in Table 3. Statistical analysis indicated no significant differences in nuclear DNA content among the cultivars. Chromosome counts revealed that all cultivars were diploid with 2*n* = 36. Chromosomes were small, generally less than 5 µm in length (Figure 2D–F).

### 3.2. Comparative Anatomy

#### 3.2.1. Leaf Microanatomy

**Figure 3:**
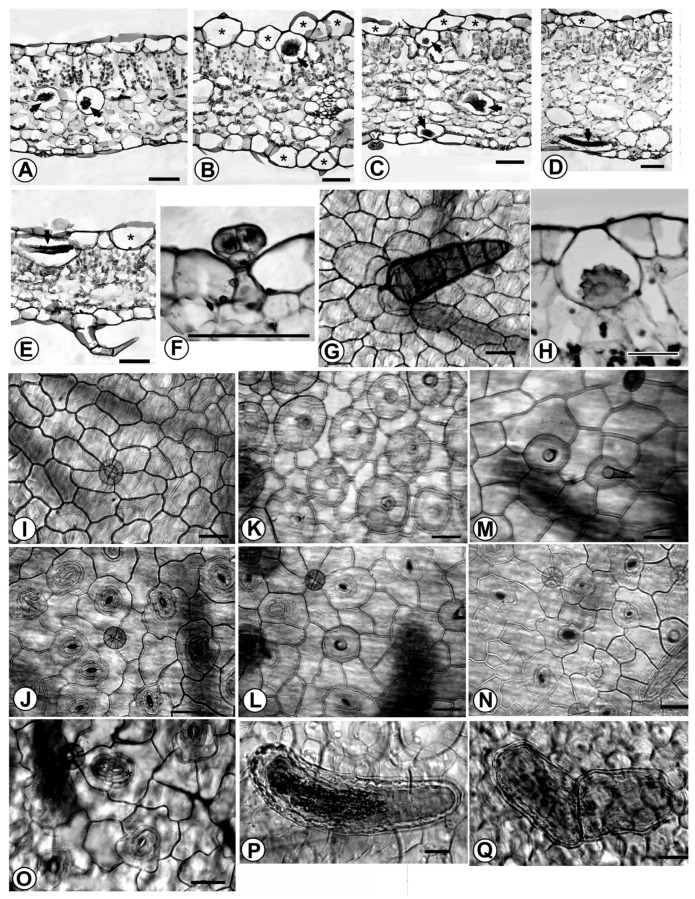
Leaf anatomy of *Fittonia* cultivars. Scale bar = 50 μm. (**A**–**E**) Transverse sections (TS) of leaves; asterisks indicate bubble cells and arrows indicate cystoliths. (**A**) Cultivar Titanic, showing flat epidermal cells and cystoliths located between the palisade and spongy mesophyll. (**B**–**E**) Leaf transverse sections of cultivars White Anne (**B**), Red Vein (**C**), Fortissima (**D**), and Black Star (**E**). (**F**) Capitate hairs of ‘Mini-Josan’. (**G**) Coniform hairs of ‘Angel Snow’, showing a complex trichome base. (**H**) Cystolith of ‘Red Star’, showing the internal cystolith crystal. (**I**,**J**) Upper and lower epidermis of ‘Titanic’, showing flat epidermal cells. (**K**,**L**) Upper and lower epidermis of ‘White Anne’, showing dense bubble cells on the upper cuticle and scattered bubble cells on the lower cuticle. (**M**,**N**) Upper and lower cuticle of ‘Jocmita’. (**O**) Lower cuticle of ‘Frankie’; the asterisk indicates contiguous stomata. (**P**) Simple cystolith of ‘Red Anne’. (**Q**) Double cystolith of ‘Leather Leaf’.

Leaf anatomical characters provided useful information for interpreting variation patterns at low taxonomic levels [24,25]. All *Fittonia* cultivars shared several features: leaves were typical C_3_ leaves with well-developed palisade and spongy mesophyll layers (Figure 3A–E).

#### 3.2.2. Leaf Epidermis

The epidermis of all cultivars consisted of a single layer of thin-walled, irregularly shaped cells lacking chloroplasts, except in the guard cells (Figure 3A–E). Intercostal epidermal cells on both surfaces were irregularly polygonal, with straight to sinuous anticlinal walls (Figure 3I–N).

Marked variation in cuticular cell morphology was observed. ‘Titanic’ and ‘Angel Snow’ exhibited flat, irregular polygonal cuticle cells (Figure 3A,I,J). In contrast, the remaining cultivars possessed distinctive, large, globose bubble-like epidermal cells scattered among flat epidermal cells. These convex cells were either obtuse or bore a small beak composed of one to three stacked cells (Figure 3B–E,K–N). Bubble-like cells were primarily distributed on the adaxial epidermis, with only sparse occurrence on the abaxial surface (Figure 3L,N). However, in ‘Superba’, ‘Red Star’, and ‘Red Angel’, numerous bubble-like cells were also present on the abaxial epidermis.

Bubble-like cells ranged from 45.5 to 101.3 µm in diameter (Figure 3K). Their density on the adaxial surface varied widely among cultivars, typically from 50 to 90 cells mm^−2^. Extreme values included as few as 18 cells mm^−2^ in ‘Frankie’ and ‘Jacmita’ and as many as 114 cells mm^−2^ in ‘White Anne’.

#### 3.2.3. Stomata

All stomata were of the diacytic type and were predominantly confined to the abaxial epidermis, with only occasional stomata on the adaxial surface. Stomatal size ranged from 12.2–23.3 to 23–30 µm (Figure 3J,L,M). Stomatal densities were highest in ‘Titanic’ (104 mm^−2^) and ‘Angel Snow’ (94 mm^−2^), whereas the remaining cultivars exhibited lower densities ranging from 45 to 87 mm^−2^ (Table S3).

Abnormal stomata were observed in ‘Frankie’, Red Anne’, ‘Red Vein’, ‘White Anne’, ‘Leather Leaf’, ‘Superba’, and ‘Jacmita’, including stomata with only one or poorly developed guard cells and occasional pairs of contiguous stomata joined at the polar ends (Figure 3O).

#### 3.2.4. Trichomes

Four types of trichomes were identified. (1) Acicular hairs were uniseriate, multicellular, and nonsecretory (Figure 3E). They occurred mainly along the leaf margins and costal regions, and their bases consisted of one or two cells. (2) Capitate glandular hairs consisted of a two-celled stalk, a four- to eight-celled head, and a single-celled base (Figure 3F), and were distributed randomly on both leaf surfaces. (3) Coniform hairs were thick, multicellular, uniseriate structures, usually composed of four to six cells. They were highly lignified, ornamented with dense spines, and had a compound base attached to five to seven surrounding epidermal cells (Figure 3G). (4) Papillae or beak-like hairs consisted of one to three stacked cells and were restricted to the apex of bubble-like epidermal cells (Figure 3B–D,K–N); they were occasionally observed at the junctions between adjacent bubble cells. Capitate glandular hairs and acicular hairs were present in all cultivars. Coniform hairs were observed only in ‘Titanic’ and ‘Angel Snow’, whereas papillae or beak-like hairs occurred in all other cultivars but were absent from ‘Titanic’ and ‘Angel Snow’.

#### 3.2.5. Cystoliths

Cystoliths were generally located in intercostal regions of the leaf. Their shapes varied from oval and oblong to curved, virgulate forms. In ‘Titanic’ and ‘Angel Snow’, most cystoliths were curved virgulate (Figure 3P). In the remaining cultivars, cystolith morphology was more variable. Double cystoliths were occasionally observed in ‘Red Vein’, ‘Red Angel’, ‘Leather Leaf’, ‘Jacmita’, ‘Superba’, ‘Black Star’, and ‘Frankie’ (Figure 3Q). In ‘Titanic’ and ‘Angel Snow’, cystoliths were restricted to the region between the palisade and spongy mesophyll layers (Figure 3A). In contrast, in the remaining cultivars, cystoliths were distributed between the palisade and spongy layers and also occurred in both epidermal layers (Figure 3B–E,H).

### 3.3. Leaf Architecture

**Figure 4:**
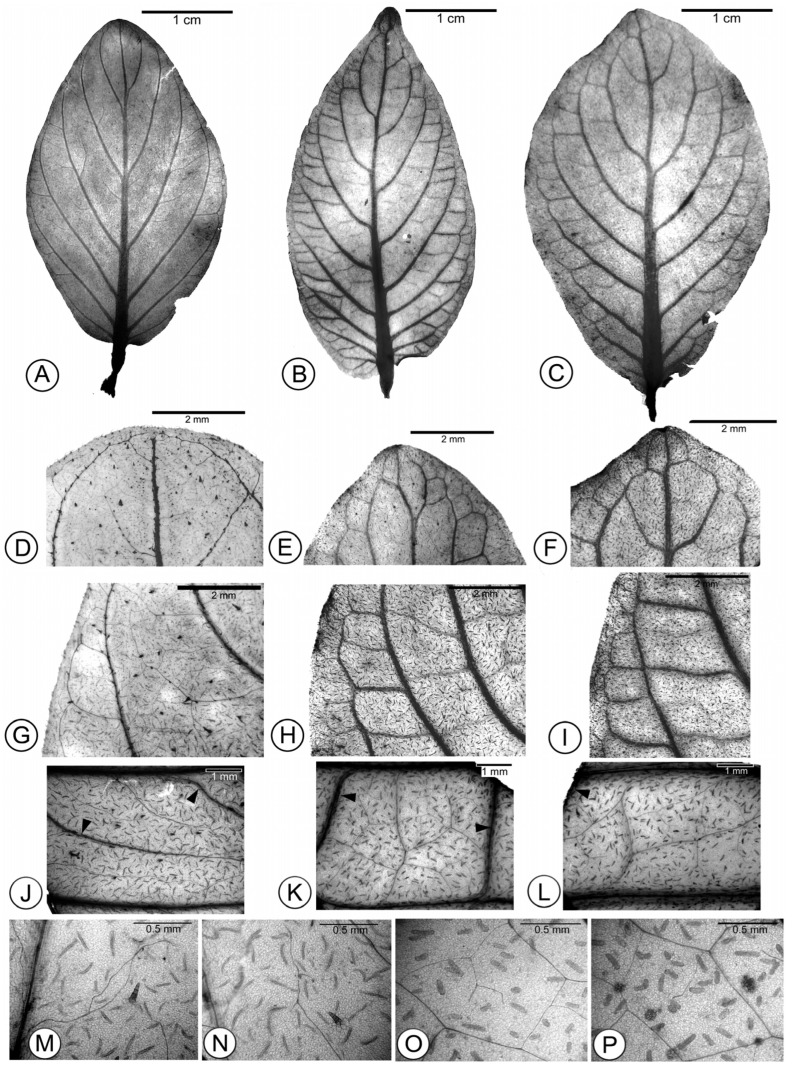
Leaf venation of *Fittonia* cultivars. (**A**–**C**) Whole-leaf venation patterns of cultivars ‘Titanic’ (**A**), ‘Black Star’ (**B**), and ‘White Anne’ (**C**). (**D**–**F**) Leaf apices of cultivars ‘Titanic’ (**D**), ‘Black Star’ (**E**), and ‘White Anne’ (**F**). (**G**–**I**) Eucamptodromous venation in *Fittonia* cultivars. (**G**) Cultivar ‘Titanic’; the arrow indicates an extremely slender, curved, randomly branched tertiary vein. (**H**,**I**) Cultivars ‘Black Star’ and ‘White Anne’, respectively; arrows indicate thick tertiary veins extending across two adjacent secondary veins. (**J**–**P**) Higher magnification of leaf venation. (**J**) Cultivar ‘Titanic’; the arrow indicates a tertiary vein extending toward the leaf margin and branching randomly into quaternary veins. (**K**,**L**) Cultivars ‘Black Star’ and ‘White Anne’, respectively; arrows indicate cross-connecting tertiary veins. In the center, quaternary veins branch irregularly to form the areolation. (**M**) Poorly developed areolation in ‘Titanic’. (**N**) Randomly branched tertiary veins in ‘Titanic’. (**O**,**P**) Areoles of ‘Black Star’, showing an unbranched veinlet (**O**) and a once-branched veinlet (**P**), respectively.

Leaves of *Fittonia* cultivars were decussate. Leaf shape was lanceolate, elliptic, or ovate (Figure 4A–C). Except for the rounded, convex leaf apex in ‘Titanic’ (Figure 4D) and ‘Angel Snow’, the leaf apex of the remaining cultivars was broadly acute with a shallow retuse (Figure 4D–F). The leaf base was shallowly cordate, truncate, or obtuse, and the leaf margin was entire or pleated.

Venation in *Fittonia* was pinnate eucamptodromous (Figure 4A–C,G–I). Secondary veins were usually alternate (Figure 4B,C) and rarely opposite (only in ‘Titanic’ [Figure 4A] and ‘Angel Snow’). Secondary vein spacing decreased toward the base or was irregular (Figure 4A–C).

In ‘Titanic’ and ‘Angel Snow’, tertiary veins were extremely slender and formed a random reticulate pattern toward the leaf margin. Tertiary veins rejoined other tertiary or secondary veins at variable angles. Their course and angle were highly variable and ramified (Figure 4G,J), resulting in absent or rare areolation (Figure 4M,N).

In contrast, the tertiary veins in the remaining *Fittonia* cultivars were prominent and crossed directly between adjacent secondary veins (Figure 4H,I), corresponding to the opposite percurrent type. The angles of the tertiary veins became increasingly obtuse away from the midrib (Figure 4B,C). Fourth-order veins branched freely and conformed to the dichotomizing type (Figure 4K,L). Due to the frequent presence of prominent tertiary veins, areolation was moderately developed (Figure 4K,L). Unbranched or once-branched freely ending ultimate veins (FEVs) were commonly observed in these cultivars, except in ‘Titanic’ and ‘Angel Snow’ (Figure 4O,P).

### 3.4. Stem and Petiole Anatomy

**Figure 5:**
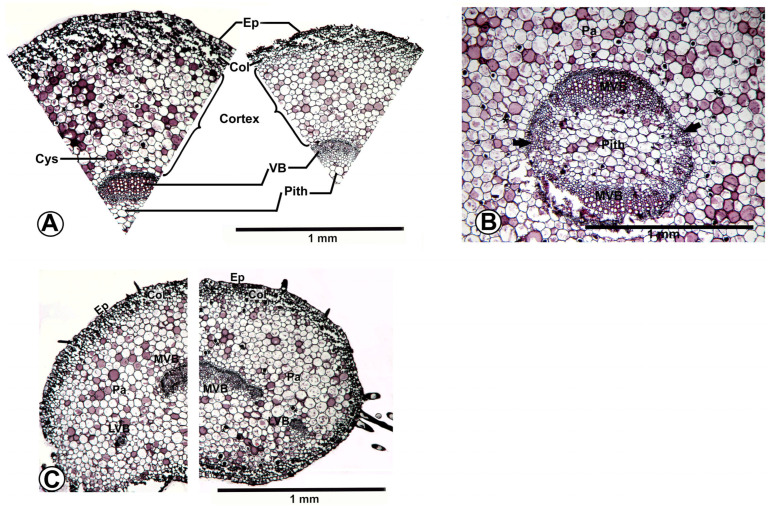
Stem and petiole transverse sections (TS) of cultivated *Fittonia*. (**A**) Stem transverse sections. Left: cultivar ‘Leather Leaf’; right: cultivar ‘Angel Snow’. Ep, epidermis; Col, collenchyma; Cys, cystolith; VB, vascular bundle. (**B**) Stem transverse section of cultivar ‘Red Anne’, showing a concentric ring of vascular bundles with two main well-developed vascular bundles (MVB) and smaller lateral vascular bundles (arrows). Cystoliths (asterisks) are scattered in the cortical and pith parenchyma. (**C**) Petiole transverse sections. Left: cultivar ‘Titanic’; right: cultivar ‘Mini-Josan’. Ep, epidermis; Col, collenchyma; Pa, cortical parenchyma; MVB, main vascular bundle; LVB, lateral vascular bundle.

Stems of *Fittonia* were not typical dicotyledonous stems (Figure 5A). A layer of collenchyma was located immediately beneath the epidermis. Inside the collenchyma, a well-developed cortex occupied most of the stem. The vascular bundles formed a distinct ring surrounding a central pith (Figure 5A,B).

The arrangement of the vascular bundle ring showed some variation. In ‘Red Angel’, ‘Mini-Josan’, ‘Titanic’, and ‘Angel Snow’, the vascular bundle ring was polarized, with two main vascular bundles formed oppositely and poorly developed vascular bundles in other parts of the ring. In large-leaved cultivars (e.g., Red Vein’, ‘Red Anne’, ‘White Anne’, ‘Superba’, and ‘Leather Leaf’), vascular bundles were well developed throughout the ring. Another notable feature was the distribution of cystoliths in the stem. Cystoliths were commonly observed in cultivated *Fittonia*, except in ‘Titanic’ and ‘Angel Snow’ (Figure 5C).

### 3.5. Diagnostic Morphological Characters Used in Traditional Taxonomy

The shape and size of inflorescence bracteoles, leaf venation color, petiole length, and plant habit are commonly used diagnostic characters in the taxonomy of *Fittonia*. Bracteoles were generally roundish, ovate, or oblong-ovate, except in ‘Angel Snow’, in which they were lanceolate. Inflorescences were not observed in ‘Titanic’ in this study. Bracteole size varied from 7 to 11 mm in length and 5 to 9 mm in width. Petiole length ranged from 0.5 to 6.2 cm, and leaf blade size ranged from 1.4 to 4.8 cm in width and 2.5 to 8.5 cm in length. Variation in bracteole size, petiole length, and leaf blade size was continuous among cultivars, without clear discontinuities.

Venation color and leaf margin type showed marked diversification. Red venation occurred in ‘Red Angel’, ‘Red Star’, ‘Red Anne’, ‘Leather Leaf’, and ‘Black Star’; white venation in ‘Titanic’, ‘Angel Snow’, ‘White Anne’, and ‘Superba’; and pink venation in ‘Frankie’, ‘Mini-Josan’, and ‘Jacmita’. Flat leaf margins occurred in ‘Titanic’, ‘Angel Snow’, ‘Red Angel’, and ‘Mini-Josan’, whereas pleated margins were typical of ‘Frankie’, ‘Red Anne’, and ‘White Anne’. Both venation color and leaf margin type were biform in ‘Red Anne’, ‘Red Star’, ‘White Anne’, and ‘Superba’. All anatomical features and traditional morphological diagnostic characters are summarized in Table S3.

### 3.6. Evolutionary Pattern of Morphological Traits

**Figure 6:**
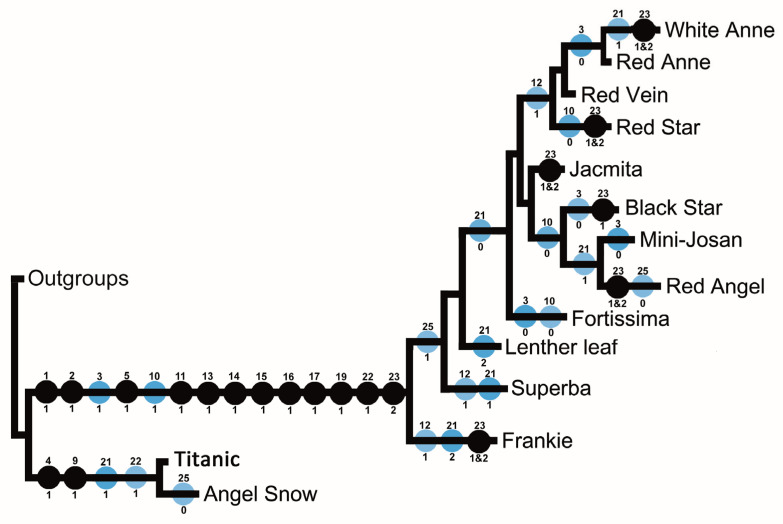
Fourteen anatomical characters observed in this study and four taxonomical characters used by DeWolf [1] and Brummitt [2,3] were mapped onto an AFLP/MSAP-based tree using Mesquite 2.75 [30]. Black solid circles indicate the plesimorphism/synapomorphism/autapomorphism; Blue transparent circles indicate the paraphyletic status of those features.

We compared 25 characters (Table S3) among 14 cultivated *Fittonia* cultivars. Features invariant across Acanthaceae and fragmented characters were excluded. Twenty characters were amenable to character-state delimitation and were mapped onto the AFLP and MSAP clustering tree using parsimony criteria (Figure 6). A total of 68 steps were required. Putative synapomorphies for Clade I (‘Titanic’ + ‘Angel Snow’) included characters 4 and 9: coniform hairs and high stomatal density. Eleven anatomical characters (1, 2, 5, 11, 13, 14, 15, 16, 17, 19, and 22) and one macromorphological character (23, plant shape) were consistent features associated with Clade II (the remaining 12 cultivars). Characters 3 and 10 (presence of double cystoliths and contiguous stomata), as well as traditional taxonomic characters 12, 21, 22, 23, and 25 (leaf margin, leaf venation color, petiole length, and bracteole size), were homoplastic.

## 4. Discussion

*Fittonia* has gained substantial horticultural importance because of its attractive foliar variegation, ease of propagation, and adaptability to interior environments [4,31]. Yet, despite its commercial prominence, the systematic relationships among cultivated forms have remained poorly resolved. The principal obstacle has been the long-standing reliance on conspicuous ornamental traits, such as leaf vein color, leaf size, and petiole length, which are likely evolutionarily labile and subject to repeated selection. Our results show that this reliance has obscured a deeper and more biologically meaningful pattern of differentiation within cultivated *Fittonia*.

A central outcome of this study is the consistent recovery of two strongly supported clusters among the 14 cultivars examined: Clade I, comprising ‘Titanic’ and ‘Angel Snow’, and Clade II, comprising the remaining 12 cultivars. The substantial AFLP band divergence between these clades indicates broad genomic differentiation, while the corresponding separation in MSAP profiles suggests that lineage divergence also extends to epigenetic patterning. Taken together, these data indicate that cultivated *Fittonia* does not constitute a single homogeneous gene pool but rather comprises two well-differentiated clusters.

Although the use of AFLP in phylogenetic inference has been debated due to potential issues of homology and homoplasy, AFLP remains highly informative for closely related taxa, recent radiation, and species complexes when large numbers of fragments are analyzed [32,33,34]. In the present study, the large number of fragments generated and the strong congruence between AFLP and MSAP topologies increase confidence that the observed pattern reflects genuine genetic and epigenetic differentiation rather than analytical artifact. More importantly, the molecular groupings are independently corroborated by a suite of stable anatomical characters, which provides a mechanistic bridge between genomic divergence and phenotype.

The anatomical differences between the two clades are not superficial. Clade I is characterized by flat cuticle cells, restricted cystolith distribution between palisade and spongy mesophyll, coniform hairs, decreasing secondary vein spacing toward the leaf base, slender, randomly reticulate tertiary veins near the margin, poorly developed or absent areolation, and absence of stem cystoliths. In contrast, Clade II possesses bubble-like epidermal cells, a broader distribution of cystoliths in leaves, papillae or beak-shaped hairs, irregular spacing of secondary veins toward the leaf base, prominent tertiary veins that cross directly between adjacent secondary veins, more developed areolation, and scattered stem cystoliths. These characters involve epidermal differentiation, mineral body deposition, trichome ontogeny, and venation architecture, all of which are developmentally integrated aspects of leaf and stem construction. Because such traits arise through coordinated developmental programs rather than simple surface variation, their concordance with the molecular clusters suggests that the observed group reflects stable biological differentiation rather than environmentally induced phenotypic plasticity alone.

This point is important for interpreting taxonomic patterns. Traditional classifications in *Fittonia* emphasize leaf venation color, petiole length, blade size, and bracteole dimensions. Our character-mapping analysis shows that these conspicuous features are homoplastic, paraphyletic, or continuously varying. Mechanistically, that pattern is plausible: venation color and foliage variegation are likely controlled by relatively few regulatory pathways affecting pigment accumulation, chlorophyll distribution, or epidermal visibility of vascular tissues, and such pathways can evolve repeatedly or be strongly modified by horticultural selection. Similarly, organ size traits such as leaf dimensions and petiole length are quantitative and developmentally plastic, making them especially vulnerable to convergence under cultivation. In contrast, micromorphological and architectural traits, including trichome morphology and tertiary venation pattern, areolation, and cystolith placement, appear to be more developmentally canalized and therefore more reliable indicators of shared ancestry.

The results also clarify why previous taxonomic treatments have been difficult to reconcile. Brummitt’s [3] separation of the “Argyroneura” and “Verschaffeltii” groups was largely based on venation color, but our data show that venation color cuts across deeper lineage structure. Likewise, Ahmad’s [9] report that bubble-like epidermal cells were restricted to var. *pearsei* conflicts with our observation that this feature is shared across all examined members of Clade II despite wide variation in vein coloration. This suggests that earlier taxonomies may have grouped cultivars by conspicuous ornamental phenotype while overlooking anatomically conserved characters that better reflect evolutionary relationships.

At the same time, our data do not allow unequivocal assignment of the two clusters to currently recognized species, particularly given that our sampling is restricted to cultivated materials. This is not because the clades are weakly differentiated, but because the historical species circumscriptions themselves appear unstable and partly inconsistent with the character combinations observed here. ‘Titanic’ and ‘Angel Snow’ share some traits with descriptions of *F. gigantea* or var. *argyroneura*, whereas Clade II shares certain features with concepts associated with var. *pearsei* or *verschaffeltii*. However, none of these matches is complete. For example, the previously reported triploid chromosome number for ‘Pearcei’ conflicts with the universal diploidy observed in the present study, and reported trichome characters do not fully align with our results. These inconsistencies suggest that the current nomenclatural framework may be based on taxonomic entities that were incompletely circumscribed, horticulturally mixed, or historically interpreted through different sets of characters.

The cytological results help refine this interpretation. All 14 cultivars were diploid (2*n* = 36) and had relatively similar genome sizes, indicating that the two clades are not separated by gross ploidy differences or major genome expansion. This suggests that the divergence between clades is more likely attributable to structural genomic differentiation, allelic divergence, and regulatory change than to polyploidization. The relatively narrow range of genome size also supports the view that cultivated *Fittonia* has a constrained genomic background despite considerable visible phenotypic diversity.

That contrast between limited cytological variation and pronounced foliar diversity is particularly noteworthy. One explanation is that a substantial proportion of cultivar differentiation is driven by relatively small genomic changes that disproportionately affect visible leaf traits. AFLP indicates appreciable DNA-level variation, whereas MSAP profiles point to differences in methylation status among cultivars and between clades. Because DNA methylation can alter gene expression without altering coding sequences, epigenetic divergence provides a plausible mechanistic basis for the observed diversification patterns in vein pigmentation, variegation, leaf texture, and related ornamental traits. Transposable element dynamics may further contribute to this process by generating both sequence polymorphism and methylation-associated regulatory variation. In a clonally propagated ornamental genus such as *Fittonia*, where sports and somatic variants can be fixed and maintained, these mechanisms could readily produce substantial phenotypic diversification from a relatively narrow underlying genome pool.

The combination of narrow genome size variation, universal diploidy, substantial AFLP, and clade-associated MSAP patterns therefore suggests a hierarchical model of divergence in cultivated *Fittonia*. At one level, two distinct diploid clusters are present, reflected in stable microanatomical and architectural characters. At another level, within-lineage diversification in ornamental phenotype appears to be amplified by smaller-scale genomic and epigenetic variation, likely reinforced through clonal propagation and horticultural selection. This model explains why cultivated *Fittonia* can display marked differences in leaf coloration, margin type, and variegation while retaining relatively limited cytological divergence.

Overall, our findings indicate that the current taxonomy of *Fittonia* does not adequately capture the biological structure of cultivated material. The two clusters recovered here represent consistently supported patterns of differentiation. Yet, their relationship to named taxa remains unresolved because traditional classifications rely heavily on characters that do not track lineage history. Resolving this problem will require direct comparison with herbarium specimens, original descriptions, and—most critically—sampling of wild populations across the native range. Only then will it be possible to determine whether the cultivated clades correspond to existing species, infraspecific taxa, previously overlooked lineages, or horticultural derivatives of more complex ancestry.

## 5. Conclusions

This study provides integrative evidence that cultivated *Fittonia* comprises two distinct and well-supported diploid clusters rather than a single morphologically variable taxon. AFLP and MSAP analyses consistently separated ‘Titanic’ + ‘Angel Snow’ from the other 12 cultivars, and this molecular and epigenetic divergence was mirrored by stable differences in leaf and stem anatomy. The congruence among genomic, epigenetic, anatomical, and cytological datasets indicates that the observed clusters reflect consistent underlying differentiation rather than superficial horticultural variation.

Our results also show that many of the characters historically used to classify *Fittonia*, including leaf venation color, petiole length, and bracteole size, are homoplastic, continuously varying, or strongly influenced by parallel selection on ornamental phenotype. In contrast, less conspicuous but more developmentally integrated traits, such as trichome morphology, cystolith distribution, tertiary venation architecture, areolation, and growth habit, appear to provide a more reliable basis for taxonomic interpretation. Mechanistically, these traits likely reflect conserved developmental programs and therefore track shared ancestry more faithfully than highly selectable pigmentation and size-related characters.

All cultivars examined were diploid and exhibited only limited variation in genome size, indicating that major cytological shifts have not been the primary driver of diversification in cultivated *Fittonia*. Instead, the pronounced foliar diversity observed among cultivars likely results from a combination of modest genomic divergence and regulatory variation, with DNA methylation potentially playing an important role in generating or stabilizing ornamental phenotypes. In a clonally propagated crop, such epigenetic and developmental variation could be readily captured and maintained through horticultural selection.

Although the two clusters identified here are clearly distinct, they cannot yet be assigned with confidence to any currently recognized species within *Fittonia*. This inability reflects not weak evidence, but rather instability in the existing taxonomy itself. Future work should integrate wild and cultivated sampling, herbarium-based taxonomic reassessment, and additional molecular datasets to determine how these cultivated lineages relate to named species and to revise species boundaries within the genus on a more robust systematic basis.

## Figures and Tables

**Table 1:** Number of fragments generated by each primer pair combination and percentage of polymorphic fragments detected in 14 *Fittonia* cultivars.

Primer Pair Combinations	No. of Fragments Generated	Variable Fragments	Primer Combinations	No. of Fragments Generated	Variable Fragments
*Eco*RI + AAG/*Mse*I + CAC	53	32	*Eco*RI + AAC/*Mse*I + CTA	40	13
*Eco*RI + AGC/*Mse*I + CAC	58	37	*Eco*RI + ACC/*Mse*I + CTA	32	20
*Eco*RI + ACA/*Mse*I + CAG	54	23	*Eco*RI + ACT/*Mse*I + CTA	53	31
*Eco*RI + ACT/*Mse*I + CAG	44	27	*Eco*RI + AGG/*Mse*I + CTA	44	26
*Eco*RI + ACC/*Mse*I + CAT	94	54	*Eco*RI + AAC/*Mse*I + CTT	19	4
*Eco*RI + AGC/*Mse*I + CAT	36	24	*Eco*RI + AGG/*Mse*I + CTT	22	1
**Total fragments: 549 and total variable fragments: 292**					
**MSAP**	***Hpa*II/*Msp*I**	***Hpa*II/*Msp*I**		***Hpa*II/*Msp*I**	***Hpa*II/*Msp*I**
*Eco*RI + ACA/*Hpa*II/*Msp*I + ACT	80	34/14	*Eco*RI + AAC/*Hpa*II/*Msp*I + AAC	40	19/19
*Eco*RI + ACC/*Hpa*II/*Msp*I + ACT	58	49/47	*Eco*RI + ACG/*Hpa*II/*Msp*I + AAC	30	24/26
*Eco*RI + ACT/*Hpa*II/*Msp*I + ACT	80	60/77	*Eco*RI + AAC/*Hpa*II/*Msp*I + AAT	50	18/22
*Eco*RI + AGG/*Hpa*II/*Msp*I + ACT	60	23/10	*Eco*RI + ACA/*Hpa*II/*Msp*I + AAT	60	22/23
*Eco*RI + AAC/*Hpa*II/*Msp*I + ACT	41	29/20	*Eco*RI + ACG/*Hpa*II/*Msp*I + AAT	70	45/34
*Eco*RI + ACG/*Hpa*II/*Msp*I + ACT	34	18/32	*Eco*RI + ACT/*Hpa*II/*Msp*I + AAT	64	23/50
**Total fragments: 667 and total *Hpa*II/*Msp*I: 265/212**					

**Table 2:** Types of MSAP bands in 14 *Fittonia* cultivars.

Code	Cultivars	Type I		Type II	Type III	Type IV	Total Amplified Bands	Total Methylated Bands	MSAP %	Full Methylated Bands	Fully Methylated Ratio%
		Total	Methylation Sensitive								
1	Frankie	213	101	210	155	90	668	556	83.23	300	44.91
2	Titanic	208	68	181	144	135	668	528	79.04	316	47.31
3	White Anne	207	92	213	149	99	668	553	82.78	312	46.71
4	Red Anne	213	90	211	157	87	668	545	81.59	298	44.61
5	Fortissima	207	92	202	164	95	668	553	82.78	297	44.46
6	Angel Snow	221	66	194	144	108	667	512	76.76	302	45.28
7	Red Star	204	88	216	154	94	668	552	82.63	310	46.41
8	Black Star	203	87	215	158	92	668	552	82.63	307	45.96
9	Red Vein	211	90	213	159	85	668	547	81.89	298	44.61
10	Mini-Josan	210	94	210	157	91	668	552	82.63	301	45.06
11	Leather Leaf	211	99	212	157	87	667	555	83.21	299	44.83
12	Jacmita	208	96	212	151	98	669	556	83.23	310	46.34
13	Red Angel	210	93	209	156	91	668	528	79.04	301	45.06
14	Superba	200	95	209	156	104	669	553	82.78	313	46.79

Note: Total methylated bands = Methylation sensitive (Type I) + Type II + Type III + Type IV. MSAP% = total methylated bands/Total Amplified bands. Fully methylated Ratio% = Type IV/Total Amplified Bands. The codes denote the cultivars included in the initial collection.

**Table 3:** Nuclear DNA contents determined by flow cytometry analysis of 14 *Fittonia* cultivars.

Code	Cultivar Name	2C-Value ± S.D. (pg)	DNA Index	1C Genome Size (Mbp)	Ploidy Level
9	Red Anne	2.92 ± 0.06	0.32	1428	2
11	Leather Leaf	2.89 ± 0.08	0.32	1413	2
5	Fortissima	2.88 ± 0.06	0.32	1411	2
13	Red Angel	2.86 ± 0.04	0.31	1398	2
10	Mini Josan	2.89 ± 0.04	0.32	1414	2
14	Superba	2.85 ± 0.06	0.31	1395	2
12	Jacmita	2.86 ± 0.01	0.31	1400	2
8	Black Star	2.94 ± 0.05	0.32	1440	2
7	Red Star	2.88 ± 0.04	0.32	1410	2
3	White Anne	2.87 ± 0.03	0.32	1404	2
1	Frankie	2.91 ± 0.04	0.32	1422	2
4	Red Vein	2.86 ± 0.05	0.31	1397	2
2	Titanic	2.74 ± 0.02	0.30	1340	2
6	Angel Snow	2.73 ± 0.03	0.30	1336	2

The codes denote the cultivars included in the initial collection.

## Data Availability

All data generated or analyzed during this study are included in this published article.

## Supplementary Materials

The following supporting information can be downloaded at: https://www.mdpi.com/article/10.3390/plants15091391/s1, Table S1. Plant materials used in AFLP, MSAP, cytological, and leaf anatomical analyses. The vouchers were kept in FLAS. Table S2. The sequences of adapters used for AFLP and MSAP analyses. Table S3. Comparison of Leaf anatomical features and traditional taxonomy features of *Fittonia* cultivars (the character status was scored for AFLP cladogram-based character mapping). Figure S1. DNA-methylation profiles using the primer combination of H/M-AAC + E-AAC.
