# Supplementary material for: Integrative Molecular, Cytological, and Anatomical Analyses Reveal Two Distinct Clusters in Fittonia Cultivars
Source: Plants (Basel). 2026 May 1;15(9):1391. doi: 10.3390/plants15091391 (PMC13165445; doi:10.3390/plants15091391)
Supplement: Supplementary file 1 [file plants-15-01391-s001.zip › plants-4241639-supplementary.pdf]

Supplementary Materials

Integrative Molecular, Cytological, and Anatomical Analyses Reveal Two Distinct Clusters in *Fittonia* Cultivars

Min Deng [1,2], Qiansheng Li [1,3] and Jianjun Chen [1,*]

Table S1. Plant materials used in AFLP, MSAP, cytological, and leaf anatomical analyses. The vouchers were kept in FLAS

Genus	Names of Species and/or Cultivars	Abbreviation	Vouchers
**Outgroups**			
Aphelandra R. BR	*A. squarrosa* Nees 'Dania'	Aphelandra	DM-F15
Hypoestes Sol. ex Br.	'Pink Splash'	Hypoestes	DM-F16
**Ingroups**			
Fittonia Coem	'Frankie'	Frankie	DM-F1
	'Titanic'	Titanic	DM-F2
	'White Anne'	White Anne	DM-F3
	'Red Anne'	Red Anne	DM-F4
	'Fortissima'	Fortissima	DM-F5
	'Angel Snow'	Angel Snow	DM-F6
	'Red Star'	Red Star	DM-F7
	'Black Star'	Black Star	DM-F8
	'Red Vein'	Red Vein	DM-F9
	'Mini-Josan'	Mini Josan	DM-F10
	'Lenther Leaf'	Lenther Leaf	DM-F11
	'Jacmita'	Jacmita	DM-F12
	'Red Angle'	Red Angle	DM-F13
	'Superba'	Superba	DM-F14

[z] The vouchers were kept in the Florida Museum of Natural History (FLAS).

Table S2. The sequences of adapters used for AFLP and MSAP analyses.

	Sequences
Adapters	
*Eco*RI-adapter I	5′-AAT TGG TAC GCA GTC TAC-3′
*Eco*RI-adapter II	5'-CTC GTA GAC TGC GTA CC-3′
*Mse*I-adatper I	5′-TAC TCA GGA CTC AT-3′
*Mse*I-adapter II	5′-GAC GAT GAG TCC TGA G-3′
*Hpa*II/*Msp*I-adatper I	5′-GAT CAT GAG TCC TGC T-3′
*Hpa*II/*Msp*I-adatperII	5′-CGA GCA GGA CTC ATG A-3′
Preselective primers	
*Eco*RI + A	5′-GAC TGC GTA CC AAT TC A-3′
*Mse*I + C	5′-GAT GAG TCC TGA GTA AC-3′
*Hpa*II/*Msp*I + 0	5'-GAT GAG TCT AGA A-CGG-3'
Selective primer pair combinations used in AFLP	
*Eco*RI + AAG / *Mse*I + CAC	*Eco*RI + AAC / *Mse*I + CTA
*Eco*RI + AGC / *Mse*I + CAC	*Eco*RI + ACC / *Mse*I + CTA
*Eco*RI + ACA / *Mse*I + CAG	*Eco*RI + ACT / *Mse*I + CTA
*Eco*RI + ACT / *Mse*I + CAG	*Eco*RI + AGG / *Mse*I + CTA
*Eco*RI + ACC / *Mse*I + CAT	*Eco*RI + AAC / *Mse*I + CTT
*Eco*RI + AGC / *Mse*I + CAT	*Eco*RI + AGG / *Mse*I + CTT
Selective primer pair combinations used in MSAP	
*Eco*RI + ACA / *Hpa*II/*Msp*I + ACT	*Eco*RI + AAC / *Hpa*II/*Msp*I + AAC
*Eco*RI + ACC / *Hpa*II/*Msp*I + ACT	*Eco*RI + ACG / *Hpa*II/*Msp*I + AAC
*Eco*RI + ACT / *Hpa*II/*Msp*I + ACT	*Eco*RI + AAC / *Hpa*II/*Msp*I + AAT
*Eco*RI + AGG / *Hpa*II/*Msp*I + ACT	*Eco*RI + ACA / *Hpa*II/*Msp*I + AAT
*Eco*RI + AAC / *Hpa*II/*Msp*I + ACT	*Eco*RI + ACG / *Hpa*II/*Msp*I + AAT
*Eco*RI + ACG / *Hpa*II/*Msp*I + ACT	*Eco*RI + ACT / *Hpa*II/*Msp*I + AAT

Table S3. Comparison of Leaf anatomical features and traditional taxonomy features of *Fittonia* cultivars (The character status was scored for AFLP cladogram-based character mapping).

Leaf anatomy features

(1) Upper cuticle: flat, without bubble cells (0); with bubble (1)
(2) Distribution of the cystoliths on cuticles: Absent on both cuticles (0); Present among both cuticle cells (1)
(3) Presence of double cystoliths: Absent (0); Present (1)
(4) Presence of the coniform hair: Absent (0); Present (1)
(5) Presence of the Papillae or stocked cells: Absent (0); Present (1)
(6) Everage density of the bubble cells (/mm^2)
(7) Diameter of the bubble cell.
(8) Size of the stomata (length x width)
(9) Density of the stomata (/mm^2): Low stomata density (<90 stomata/ mm^2): (0), High stomata density (> 90 stomata/ mm^2): (1)
(10) Presence of contiguous stomata: Absent (0); Present (1)
(11) Shape of leaf apex: Rounded (0); Retuse (1)
(12) Leaf margin: Flat (0); Pleated (1)
(13) Secondary vein spacing: Irregular toward base (0); Decreasing toward base (1)
(14) Secondary vein origination: Alternate (0), Almost opposite (1)
(15) Tertiary vein category: Random reticulate [tertiaries rejoin with other tertiary veins or secondary veins at random angles] (0); Almost opposite percurrent [most tertiaries cross between adjacent secondaries in parallel paths] (1)
(16) Tertiary vein course and angle variability: Inconsistent & ramified (0); Increasing immediately & straight (1)
(17) Prominence of the tertiary veins: Weakly (0); Prominent (1)
(18) Fourth vein category: Fourth vein almost not developed (0); Dichotomizing (4th branch freely and are the finest vein order the leaf exhibits) (1)
(19) Areolation: Poorly developed or no areolation (0); Moderately developed (1)
(20) The freely ending ultimate veins of the leaf: Unbranched (0); 1-branched (1)
(21) Presence of cystolith in stem and petiole: Absent (0), Present (1)

Characters applied in traditional taxonomy

(22) Leaf vein color: Reddish (0), White (1), and Pink (2)
(23) Petiole of lower leaf: Long, more than 3 cm (0); Short, less than 2 cm (1)
(24) Plant shape: totally procumbent (0); Somewhat creeping, but main stem erect (1); Erect (2)
(25) Bracts shape: Lanceolate (length: diameter > 2) (0); Roundish, ovate to oblong (length: diameter < 2) (0)

Cha. no. / *Cultivar*	*1*	*2*	*3*	*4*	*5*	*6* /mm^2	*7* (μm)	*8* (μm)	*9* /mm^2	*10*	*11*	*12*	*13*	*14*	*15*	*16*	*17*	*18*	*19*	*20*	*21*	*22*	*23*	*24*	**25**
Frankie	1	1	1	0	1	18	72.7–95.4	24.7-34.9×15.3–18.4	70 (0)	1	1	1	0	0	1	1	1	1	1	0&1	2	0	1&2	?	?
Titanic	0	0	0	1	0	—	—	30.6–35.1× 19.3–23.2	104 (1)	0	0	0	1	1	0	0	0	0	0	—	1	1	0	?	?
White Anne	1	1	0	1	1	61	70.0–101.3	26.0–37.0× 15.2–16.4	87 (0)	1	1	1	0	0	1	1	1	1	1	0&1	1	0	1&2	1	1
Red Anne	1	1	0	0	1	48	54.3–77.8	23.0–29.9×14.4–16.3	54 (0)	1	1	1	0	0	1	1	1	1	1	0&1	0	0	2	1	1
Fortissima	1	1	0	0	1	60	74.6–98.8	26.9–32.2×13.4–18.7	45 (0)	0	1	0	0	0	1	1	1	1	1	0&1	2	0	2	1	1
Angle Snow	0	0	0	1	0	—	—	31.6–35.2×17.8 –22.3	98 (1)	0	0	0	1	1	0	0	0	0	0	—	1	1	0	0	0
Red Star	1	1	1	0	1	77	45.5–67.6	24.8–32.1×12.1–18.7	64 (0)	0	1	1	0	0	1	1	1	1	1	0&1	0	0	1&2	1	1
Black Star	1	1	0	0	1	48	72.2–88.2	23.5–25.7×14.4–16.9	68 (0)	0	1	0	0	0	1	1	1	1	1	0&1	0	0	1	1	0&1
Red Vein	1	1	1	0	1	54	73.3–84.4	24.2–32.4×13.1–16.9	56 (0)	1	1	1	0	0	1	1	1	1	1	0&1	0	0	2	1	1
Mini-Josan	1	1	0	0	1	54	73.3–84.4	24.2–32.4×13.1–16.9	56 (0)	0	1	0	0	0	1	1	1	1	1	0&1	0	0	2	?	?
Lenther Leaf	1	1	1	0	1	48	72.2–88.2	23.5–25.7×14.4–16.9	68 (0)	1	1	0	0	0	1	1	1	1	1	0&1	0	0	2	1	1
Jacmita	1	1	1	0	1	18	66.56–75.5	26.2–37.9×14.3–19.9	70 (0)	1	1	0	0	0	1	1	1	1	1	0&1	0	0	1&2	1	1
Red Angel	1	1	1	0	1	65	78.6–97.4	24.3–33.5×14.2–16.7	52 (0)	0	1	0	0	0	1	1	1	1	1	0&1	2	0	1&2	1	0
Superba	1	1	1	1	1	114	70.7–95.6	27.5–33.0×13.9–15.5	79 (0)	1	1	1	0	0	1	1	1	1	1	0&1	1	0	2	1	1

* (1) "?" The characters were not detected in this study; (2) "—" did not exist.

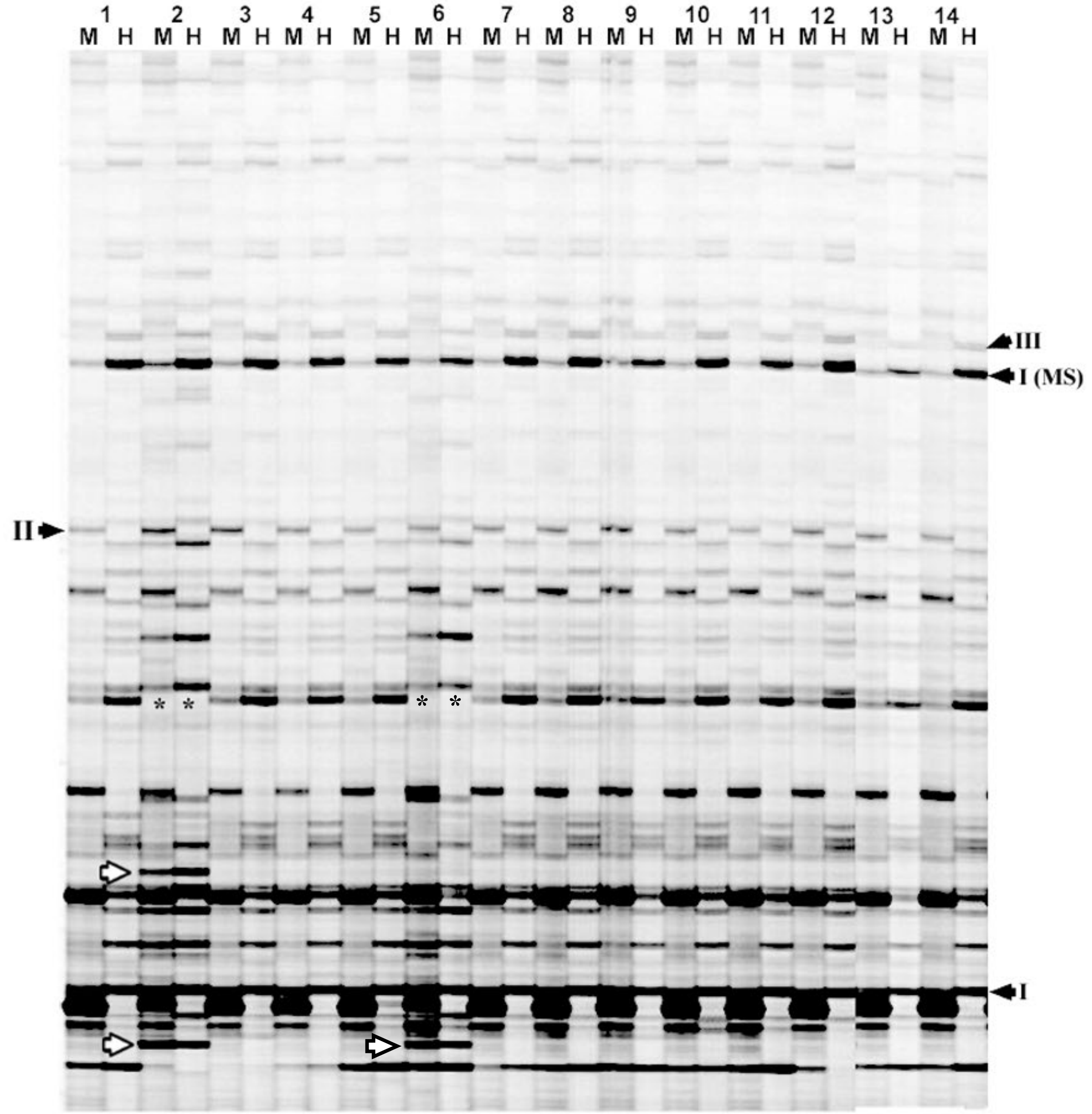

Figure S1. DNA-methylation profiles using the primer combination of H/M-AAC + E-AAC. Lane H and M correspond to two sets of restriction enzyme combinations, *Eco*RI/MspI and *Eco*RI/HpaII, respectively. Solid arrows I, I (MS), II, III indicate MSAP bands of Type I (I), Type I (methylation sensitive), Type II (II), Type III (IV). "*" indicated the absent bands both in *Eco*RI/*Hpa*II and *Eco*RI/*Msp*I (Type IV). The white blank arrow shows the cultivar-specific bands. The sample numbers for AFLP and MSP, and the corresponding cultivar names, are summarized in Table 2.
